# COVIDomic: A multi-modal cloud-based platform for identification of risk factors associated with COVID-19 severity

**DOI:** 10.1371/journal.pcbi.1009183

**Published:** 2021-07-14

**Authors:** Vladimir Naumov, Eugene Lane, Stefan Pushkov, Ekaterina Kozlova, Konstantin Romantsov, Alexander Kalashnikov, Fedor Galkin, Nina Tihonova, Anastasia Shneyderman, Egor Galkin, Arsenii Zinkevich, Stephanie M. Cope, Ramanathan Sethuraman, Tudor I. Oprea, Alexander T. Pearson, Savas Tay, Nishant Agrawal, Alexey Dubovenko, Quentin Vanhaelen, Ivan Ozerov, Alex Aliper, Evgeny Izumchenko, Alex Zhavoronkov

**Affiliations:** 1 Insilico Medicine Hong Kong Ltd, Pak Shek Kok, New Territories, Hong Kong; 2 School of Biology, Lomonosov Moscow State University, Moscow, Russia; 3 Intel Corporation, Santa Clara, California, United States of America; 4 Intel Corporation, Bangalore India; 5 Department of Internal Medicine, University of New Mexico School of Medicine, Albuquerque, New Mexico, United States of America; 6 University of New Mexico Comprehensive Cancer Center, Albuquerque, New Mexico, United States of America; 7 Autophagy Inflammation and Metabolism Center of Biomedical Research Excellence, University of New Mexico Health Sciences Center, Albuquerque, New Mexico, United States of America; 8 Department of Rheumatology and Inflammation Research, Institute of Medicine, Sahlgrenska Academy at University of Gothenburg, Gothenburg, Sweden; 9 Faculty of Health and Medical Sciences, Novo Nordisk Foundation Center for Protein Research, University of Copenhagen, Copenhagen, Denmark; 10 Department of Medicine, Section of Hematology and Oncology, University of Chicago, Chicago, Ilinois, United States of America; 11 Pritzker School of Molecular Engineering, University of Chicago, Chicago, Ilinois, United States of America; 12 Department of Surgery, Section of Otolaryngology-Head and Neck Surgery, University of Chicago, Chicago, Ilinois, United States of America; Johns Hopkins University, UNITED STATES

## Abstract

Coronavirus disease 2019 (COVID-19) is an acute infection of the respiratory tract that emerged in December 2019 in Wuhan, China. It was quickly established that both the symptoms and the disease severity may vary from one case to another and several strains of SARS-CoV-2 have been identified. To gain a better understanding of the wide variety of SARS-CoV-2 strains and their associated symptoms, thousands of SARS-CoV-2 genomes have been sequenced in dozens of countries. In this article, we introduce COVIDomic, a multi-omics online platform designed to facilitate the analysis and interpretation of the large amount of health data collected from patients with COVID-19. The COVIDomic platform provides a comprehensive set of bioinformatic tools for the multi-modal metatranscriptomic data analysis of COVID-19 patients to determine the origin of the coronavirus strain and the expected severity of the disease. An integrative analytical workflow, which includes microbial pathogens community analysis, COVID-19 genetic epidemiology and patient stratification, allows to analyze the presence of the most common microbial organisms, their antibiotic resistance, the severity of the infection and the set of the most probable geographical locations from which the studied strain could have originated. The online platform integrates a user friendly interface which allows easy visualization of the results. We envision this tool will not only have immediate implications for management of the ongoing COVID-19 pandemic, but will also improve our readiness to respond to other infectious outbreaks.

## Author summary

This article introduces COVIDomic, a new integrative multi-omics online platform designed to facilitate the analysis of the large amount of health data collected from COVID-19 patients. The COVIDomic platform includes a user-friendly interface and provides a set of bioinformatics tools for the analysis of multi-modal metatranscriptomic data to determine the origin of the coronavirus strain and the expected severity of the disease. An analytical workflow includes microbial pathogens community analysis, COVID-19 genetic epidemiology and patient stratification. These features allow studying the presence of common microbial organisms, their antibiotic resistance and the severity of the infection, as well as obtaining insights on the geographical locations from which the strain could have originated. Such openly distributed multi-modal platform will greatly accelerate the ongoing COVID-19 research and improve our readiness to respond to other infectious outbreaks.

## Introduction

Coronaviruses (CoVs) are a group of viruses that can infect the respiratory, gastrointestinal, hepatic and central nervous systems of humans, livestock, avians, bats, mice, and other wild animals [1,2]. Until recently, the few coronaviruses known to circulate in the human population caused mild respiratory infections and were regarded as relatively harmless respiratory pathogens [3]. The emergence of the severe acute respiratory syndrome coronavirus (SARS-CoV) and the Middle East Respiratory Syndrome (MERS) coronavirus demonstrated that coronaviruses can cause severe and eventually fatal respiratory tract infections in humans [4]. In December 2019, atypical pneumonia cases emerged in Wuhan (China) with clinical symptoms consistent with viral pneumonia. The investigations of the epidemiological, clinical, laboratory and radiological characteristics of infected patients, allowed to identify the cause as a novel CoV similar to SARS-CoV, named SARS-CoV-2 [5]. SARS-CoV-2 is the seventh member of the CoVs family known to infect humans, which also include 229E, NL63, OC43, HKU1, MERS-CoV and SARS-CoV. Three of these strains proved to be highly pathogenic (SARS-CoV, MERS-CoV, and SARS-CoV-2) and caused an endemic of severe CoV disease [6].

The family Coronaviridae, subfamily orthocoronavirinae, includes four genera based on their genetic properties: Alphacoronavirus, Betacoronavirus, Gammacoronavirus, and Deltacoronavirus. SARS-CoV-2 belongs to the β group of coronaviruses [7]. The coronavirus single-stranded positive-sense RNA genome is the largest among all RNA viruses (almost two folds larger than that of the second largest RNA virus) [8]. Unlike most RNA viruses, SARS-CoV-2 has a RNA polymerase proofreading capability. However, studies have shown that SARS-CoV-2 tends to have a high mutation rate [9]. These mutations can not only induce changes in virulence, infectivity, and transmissibility [10], but may also allow developing resistance to drugs and bypassing immune surveillance. While investigating the pattern and frequency of mutations is of primary importance, a comprehensive assessment of how these variants influence the fitness of a virus in a genetically diverse population requires access to a large number of viral sequences, which would allow tracing the viral genome evolution and correlating these changes with clinical symptoms and outcomes.

Like SARS-CoV, SARS-CoV-2 enters target cells through an endosomal pathway and uses the same cell entry receptor, angiotensin-converting enzyme II (ACE2) [11,12]. The fact that the virus uses receptors expressed predominantly in lungs [13,14], contributes to its very high transmissibility and spread rate. It was demonstrated that the key factor in the transmissibility of COVID-19, an infectious disease caused by SARS-CoV-2, is the high concentrations of viral particles in the upper respiratory tract, even among presymptomatic patients [15]. SARS-CoV-2 could be isolated at high concentrations from the nasal cavity or saliva of the patients even before the development of symptoms. Furthermore, a large subset of infected individuals remain asymptomatic, playing a major role in the transmission of SARS-CoV-2 [16]. As such, symptom-based screening on its own fails to detect a high proportion of infectious cases, compromising traditional infection control and public health strategies which rely heavily on the early detection of the disease to contain its spread [16].

A growing number of studies have investigated the risk factors related to the clinical outcomes in COVID-19 patients [17,18,19]. Earlier reports have established that age, increased C-reactive protein level and decreased lymphocyte numbers are potential risk factors for COVID-19 progression [20]. Among other recently revealed risk factors and comorbidities (such as coronary artery disease, hyperlipidemia, hypertension, chronic lung disease, immunodeficiency, obesity, diabetes and history of cancer), bacterial community change in a pulmonary fluid [21] and increased abundance of pathogenic microbiota [22] could also play role in disease severity and outcome. For instance, recent studies have shown that the microbiota in lungs contributed to the immunological homeostasis and potentially altered susceptibility to viral infection [23,24]. On the other hand, the lung microbiota could also be regulated by invading viruses [25]. While it is becoming apparent that both clinical and genetic risk factors may contribute to COVID-19 susceptibility and severity, the complete spectrum of risk factors associated with COVID-19 progression still remains to be established. Currently, there is no computational service that allows the aggregation of the different types of molecular data obtained from COVID-19 infected individuals together with metadata, in order to perform a comprehensive and integrative analysis.

In this article, we present COVIDomic, a novel online platform designed to support the analysis of massive amounts of genetic and health data generated during the evolution of COVID-19 pandemic. The platform provides a set of bioinformatics tools for processing of the multimodal data sets, including viral genomes, blood test results, clinicopathological data and even transcriptomic sequencing of lung fluid, their analysis and interpretation with the goal of identifying risk factors associated with severity of COVID-19 and other diseases induced by viral infections. Here we describe the platform and the methods used for its development and implementation. We next illustrate the usefulness of this platform by predicting severity of COVID-19 disease in different cohorts of patients using machine learning (ML) models trained on biochemistry and viral genome data. Feature importance algorithms were applied to identify clinical parameters and genomic mutations which can affect the severity of the COVID-19 disease in patients. Availability and constant development of the open access platforms, such as COVIDomic, which enable an integrative analysis of a wide combinations of anonymized patient data uploaded by the user together with a variety of existing data sets, will not only have immediate implications for management of the ongoing COVID-19 pandemic, but will also improve our readiness to respond to other infectious outbreaks.

## Design and implementation

### General overview

**Fig 1:**
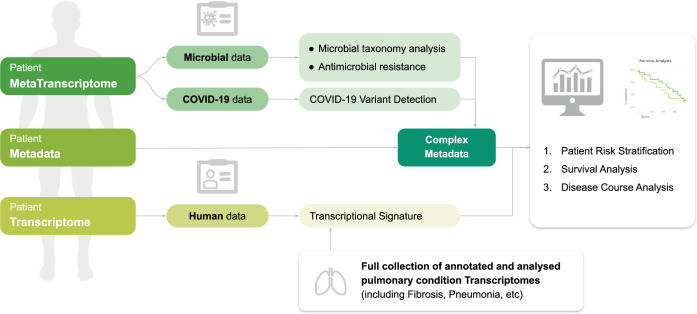
Overview of the COVIDomic platform. The user can upload transcriptomic, metatranscriptomic and clinicopathological data, which is used together with the internal data collection as an input for a comprehensive set of quantitative analyses, including patient stratification, survival, disease course and risk factor analysis. All results of the analysis can be visualized using tools provided by the platform and a comprehensive report can be downloaded by the user.

The COVIDomic platform (available at http://covidomic.com/) consists of three components (Fig 1). The first component is a web interface, which allows users to upload a wide range of genetic and clinicopathological data obtained from patient cohorts. In addition to the patient metadata which contain information about age, evolution of the disease, causes of death, etc., the platform also supports meta-transcriptomic data of the patients pulmonary fluid which provide information on the gene regulation of the microflora during the coronavirus infection.

The second component is devoted to processing of the uploaded data and bioinformatic analysis. Once a dataset is uploaded, several analytical protocols are automatically performed by the pipeline. The metatranscriptomic data of the lung tissues is used to analyze the evolution mechanisms of the pathology at the molecular level, as well as to obtain a comprehensive overview of the species diversity of the patient microbiome and its possible correlations with COVID-19 severity. The computations include a susceptibility analysis of a predefined set of antibiotics. The genome of the SARS-CoV-2 virus corresponding to each patient is assembled and used to determine the presence of potential viral genome mutations, to construct a genealogy and suggest mechanisms of propagation of the viral variants around the world.

The third component of the platform includes a set of tools that allow the user to visualize the uploaded data, to investigate the results of the analyses generated by the second component of the platform, and to perform additional post-analysis. There are three types of post-analysis available: *(i)* The combined analysis of the patient metadata, molecular changes in lung tissue, changes in species’ microflora diversity and genomic mutations of the virus; *(ii)* The assessment of statistically significant risks factors corresponding to the worst case scenario of the disease evolution; *(iii)* The possibility to test various scientific hypotheses and to cluster the set of patients into groups according to the different risk factors identified. A detailed technical description of the platform capabilities and their implementation is provided in S1 Text.

### Loading user data

**Fig 2:**
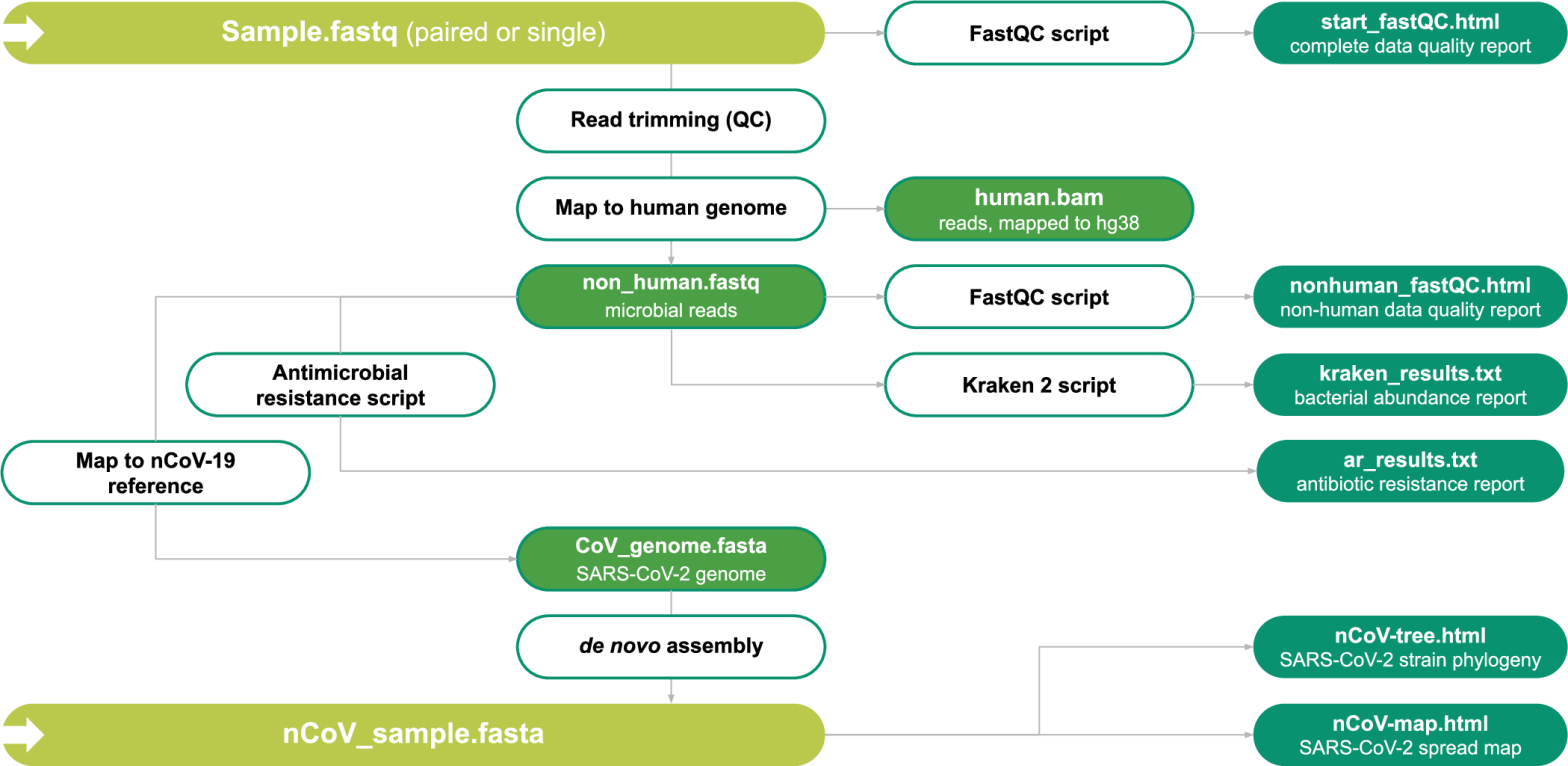
Metatranscriptome analysis for COVID-19 patients. The Schematic chart summarizes the data processing pipeline. Green boxes represent different data sets which are either used as an input or provided as output. White boxes represent the different scripts and software used to perform data processing. See main text for details.

The system includes a user friendly interface for uploading samples with their corresponding metadata. The users can upload the raw data of their metatranscriptomic samples, biochemical parameters, age and the genome sequence of the SARS-CoV-2 (if available). For a large number of samples all necessary information can be uploaded in a tabular form. Once uploaded, sequence data undergo an automatic quality control procedure performed by the FastQC software (https://www.bioinformatics.babraham.ac.uk/projects/fastqc/) (see Fig 2 for an overview of the data processing steps). The results are presented in the form of a report embedded in a web application that summarizes the quality of reads and other parameters. Upon completion of the quality control procedure, this data, along with the metatranscriptomic data and viral genomic sequences published by the scientific community, is used, as an input for further analysis as described below.

### Metatranscriptome analysis

The SARS-CoV-2 infection can cause upper respiratory tract illness, severe viral pneumonia with respiratory failure, and even death. The complete spectrum of risk factors that stimulates a severe disease course is not yet completely understood. Metatranscriptome analysis of lung fluid, nasal and oral swabs can be combined and analyzed to build a better understanding of the SARS-CoV-2 genomic variants, phylogeny, and secondary microbial pathogens that can constitute risk factors for specific groups of patients and lead to severe illness.

#### Alignment to the reference human genome.

The alignment of reads with the human reference genome is the first stage of the metatranscriptome analysis. The objective is to distinguish and separate the human reads from microbial and viral transcripts. The COVIDomic pipeline utilizes the BWA alignment program [26] (http://bio-bwa.sourceforge.net/), with the human genome build version 38 (GRCh38) used as a reference. The mapped human reads are stored as a text-based sequence alignment map (SAM) file that contains the alignment coordinates for each read in the reference genome (if any). To optimize the analysis, SAM files are converted to binary format (BAM) and then indexed using the samtools software package (http://www.htslib.org/doc/samtools.html). For the analysis of the microorganism presence, the reads that do not align to the human transcriptome are selected and stored in a separate fastq file (‘non-human.fastq’).

#### Analysis of the pathogenic bacteria diversity.

Kraken 2 [27] is a program for the diversity analysis of microorganisms present in the samples. It uses the associated database Minikraken 2, which contains the complete genome sequences for a variety of microorganisms, as a reference for aligning non-human reads (‘non-human.fastq’) generated by the BWA alignment program. The principle underlying the classification is based on a search of k-mers that correspond to the genomic sequences of the reference microorganisms. This procedure results in a list of microorganisms with their corresponding taxonomic groups (strains, species, genera, and families of microorganisms) and a number of read counts that were mapped to each of the taxa. The results obtained from various samples are then unified and combined into a matrix-like file for the subsequent analysis, and are later displayed in the form of a customizable barplot.

#### Analysis of antibiotic resistance.

The Comprehensive Antibiotic Resistance Database (CARD, https://card.mcmaster.ca/) contains sequences of genes known to provide protection against various antimicrobial agents [28]. The non-human reads (‘non-human.fastq’) are aligned with the CARD database using the BWA alignment program and processed by the samtools as described above. For each gene, this analysis yields the number of reads that have been aligned with each antibiotic resistance gene sequence, along with the position of such reads. This allows to calculate the number of reads mapped (vertical coverage) and the percentage of the gene sequence covered (horizontal coverage). The combination of these two metrics provides an indication of the expression of protective genes present in the sample. This information can be visualized as a heatmap (see the “Visualization and post-analysis” section).

#### SARS-CoV-2 de novo genome assembly.

The non-human reads (‘non-human.fastq’) are then aligned to the reference SARS-CoV-2 genome, with the mismatch threshold reduced. This allows to extract reads that are similar but not necessarily identical to the reference sequence. Selected reads are then assembled into the complete genome by the SPAdes software (http://cab.spbu.ru/software/spades/) [29]. The quality of the genome assembly is controlled with the quast software (http://cab.spbu.ru/software/quast/) [30]. The final genome sequence is stored in a fasta file format. It should be noted that the genome sequence can also be assembled and uploaded manually by the user.

#### Reconstruction of SARS-CoV-2 recent phylogeny.

Reference genomes were obtained from the National Genomics Data Center (NGDC) 2019nCoVR-2019 database. To accelerate the analysis, a preliminary alignment of the SARS-CoV-2 genome sequences is performed and followed by a precalculation of the known part of the distance matrix. Multiple preliminary alignments are performed using the MAFFT algorithm (https://mafft.cbrc.jp/alignment/software/) [31]. The resulting alignment is indexed and then modified by adding a custom sequence using the SINA program (https://github.com/epruesse/SINA) [32]. This results in a total multiple alignment, which is used to calculate the Hamming distance matrix. Then, a UPGMA radial phylogenetic tree is built for all the analyzed sequences. For the geographic analysis of the requested hCoV strain, a subtree that contains the studied sequence is extracted from the constructed tree.

### Visualization and post-analysis

#### Microbial pathogens community analysis.

The microbiome taxonomy profiling of SARS-CoV-2 infected patients can help finding possible associations between the presence of particular species and outcomes of the disease. The user receives a report summarizing the representation of bacterial taxa in a set of samples grouped by the disease. This information is presented in four different formats: (i) A PCA chart with all analyzed samples, (ii) a bar chart with percentage ratios, (iii) a heatmap showing the antibiotic resistance of the bacterial community, and (iv) a table with the possibility to rank taxa according to the average difference in percentages of bacterial composition between groups of samples and the significance of this difference. The user can also define two groups of samples and perform differential analysis to examine the possible associations of microbial presence with the disease.

**Fig 3:**
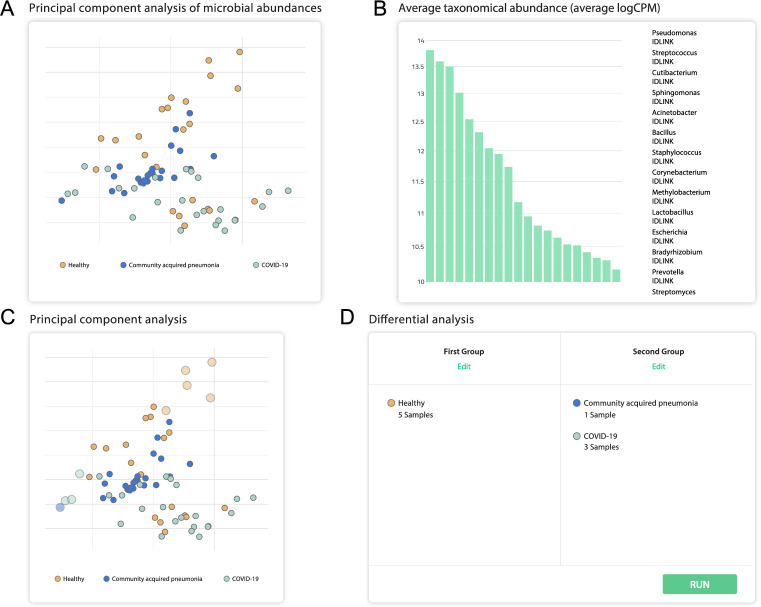
Overview of selected functionalities offered by the platform and their associated displays. These results were generated using experimental samples obtained from [33], which includes metatranscriptomic data for 8 severe and 23 non-severe COVID-19 cases. (A) PCA analysis of microbial abundance. (B) Average taxonomic abundance (X-axis: different taxa, ordered from left-to-right in the same order as in the graph legend. Y-axis: logCPM (counts per million expressed in base 10 logarithm)). (C) Interactive plot which can be used to select the samples to be used for microbial differential presence analysis. (D) Additionally, two groups can be chosen manually to perform a subsequent analysis using the deseq2 software.

**Fig 4:**
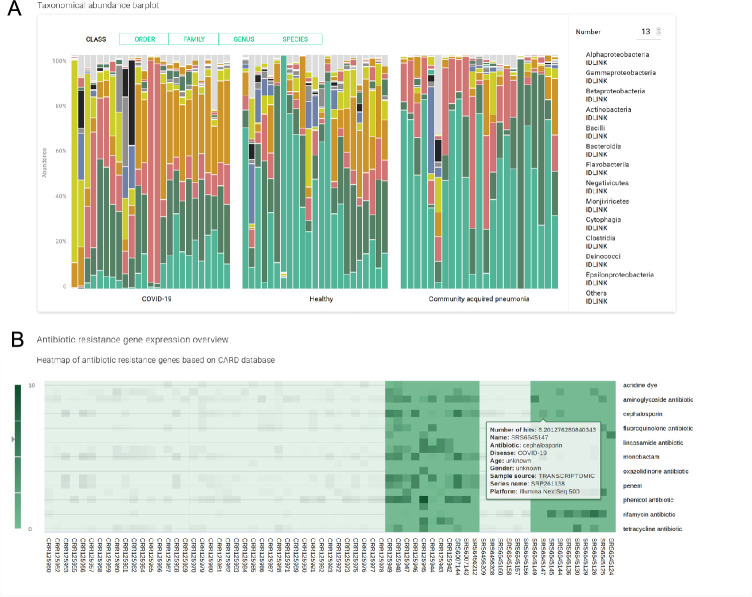
Overview of selected functionalities offered by the platform. (A) A bar plot shows the presence of the most common microbial classes, the number and level of the taxa could be specified by the user. (B) The heatmap shows the expression levels of the genes known to provide resistance to certain antibiotics. The dataset used to generate the plots was obtained from [33].

The main functionalities of the COVIDomic workflow allow the user to:

- View the uploaded data in the form of a PCA chart, with the ability to examine the presence of microbial agents for each sample (Fig 3A and 3B).
- Divide the analyzed samples into two groups and perform a differential expression analysis to find the differences in bacterial abundance of these groups (Fig 3C and 3D).
- Compare the relative abundance of every bacterial genus found, identify the correlation of the SARS-CoV-2 infection with the patient microflora, and view the similarities and the differences in comparison with other pulmonary illnesses (Fig 4A).
- Explore the levels of antibiotic resistance gene expression within the samples to evaluate the overall antibiotic resistance of the host bacterial community, and provide advice on the antimicrobial therapy to be administered (Fig 4B).
- Generate the list of the most relevant features influencing the severity of COVID-19 disease and perform risk factor analysis, whose results can be used for patient risk stratification.

#### Using the COVIDomic platform for risk factor analysis.

The workflow implemented within the COVIDomic platform allows the integrative analysis of all defined patient signatures, including microbial communities and SARS-CoV-2 mutations together with the patients metadata set that includes blood biochemical characteristics and clinicopathological characteristics. Such multi-factorial analysis provides an effective and reliable approach for patient stratification and for the identification of statistically significant risk factors. A first approach was designed to handle the patient’s clinicopathological data, while a second one was implemented for the analysis of the viral genome data.

The search for risk factors based on the patients’ blood biochemistry data and other clinical parameters is performed using logistic regression (LR) and decision tree (DT) classifiers. These two types of ML techniques are implemented in Python using the scikit-learn package [34] within the COVIDomic platform, and are trained to distinguish severe from non-severe cases.

Further steps include exploratory data analysis and results visualization. The deployment of a predictor based on features extracted from biochemistry data offers several advantages. The biochemical analysis of patient’ serum is an inexpensive and rapid procedure routinely performed in hospitals, and the output generated by ML predictors based on the features extracted from biochemistry data are easier to interpret by the clinicians. Additionally, using biochemistry data allows a comparative analysis with subgroups of healthy patient cohorts with similar age and gender distribution. Assuming that the patient data is properly recorded, it is possible to perform a longitudinal analysis of the measured biochemical indicators of a given patient before and after the onset of the disease and during the different stages of clinical progression.

Using SARS-CoV-2 genome data for the risk factor analysis presents specific challenges. Viral mutation data are high dimensional and more complex to analyze and interpret than clinical data. In the COVIDomic platform, the aligned genome data is transformed into matrix format and used to train supervised ML models. To evaluate the best pipeline, we have trained five ML models. For four of them (LR, Linear support vector machine (SVM), k-nearest neighbor (KNN) and Random Forest (RF) classifier) we used the Scikit Learn implementation, while Microsoft implementation of the Light Gradient Boosting Machine (LGBM) model was used as fifth model. The model selection was performed with 5 fold cross-validation and with plain grid search for finding the best hyperparameters for each model (LGBM hyperparameters were optimized with optuna). For each fold, 5 metrics were calculated (f1 score, precision, recall, Matthew correlation coefficient and AUC). The metric values were averaged over 5 stratified folds. The best classification results were obtained using the LGBM and LR models (F1 score of 0.77 and 0.74 respectively), which were subsequently integrated within the COVIDomic platform. Two feature importance algorithms are used to extract the interpretable information related to the contribution of the input features. The first one, a SHaply Additive exPlanations (SHAP) method, is an approach to explain individual predictions using Shapley Values, a method from coalitional game theory [35]. The second one, a Permutation Feature Importance (PFI) method, is based on the idea that shuffling the most valuable features’ values leads to a decrease in the prediction score of the model. In other words, PFI is based on the decrease in model performance. SHAP Tree Explainer and Linear Explainer were used for LGBM and Logistic regression feature importance computing respectively. The SHAP and PFI methods work under the assumption that features (i.e. mutations) are independent from each other and cannot identify significant feature combinations (i.e. that some mutations might indirectly affect other mutations). While this may result in the loss of epistasis information, we only consider individual mutation influence. In the future, the platform will be adapted to take feature combinations into account. This will include the deployment of additional computational resources, as handling such a large variety of important features with all possible combinations comes with an increase of the algorithmic complexity. Another issue that needs to be addressed to identify statistically significant feature combinations is the lack of sequenced genomes. Indeed the number of possible feature combinations is substantially higher than the amount of samples currently available. The feature importance analysis provides the list of the most important mutations associated with the disease severity. Additionally, the COVIDomic platform provides tools to identify risk factors and to predict disease severity from patient metadata, and metatranscriptome data. While using the platform for other types of input is technically possible, it requires further system adjustments which will be implemented in the coming system updates.

## Results

### Feature importance analysis based on biochemistry data

For our study, biochemical data was obtained from Shen et al. [36] dataset, containing 28 severe and 37 non-severe COVID-19 patients [37]. Using COVIDomic, we have analyzed 12 clinical measurements including white blood cell count, lymphocyte count, monocyte count, platelet count, C-reactive protein (CRP), alanine aminotransferase (ALT), aspartate aminotransferase (AST), glutamyltransferase (GGT), total bilirubin (TBIL), direct bilirubin (DBIL), creatinine, and glucose. Age of the patient was also considered, as it is recognized as an important factor for affecting COVID-19 severity [38,39].

As the initial dataset contained a relatively small number of samples, we have used the Synthetic Minority Over-sampling Technique (SMOTE) [40] implementation in Python [41] as an oversampling technique on the set of experimental biochemistry specimens to generate synthetic samples, while maintaining the internal dependencies within the data. This allows training ML models on larger datasets and provides an opportunity to assess the capabilities of the platform to handle realistic datasets consisting of a large sample number. SMOTE is commonly applied to handle imbalanced learning tasks, including disease molecular mechanisms domain [42,43].

**Fig 5:**
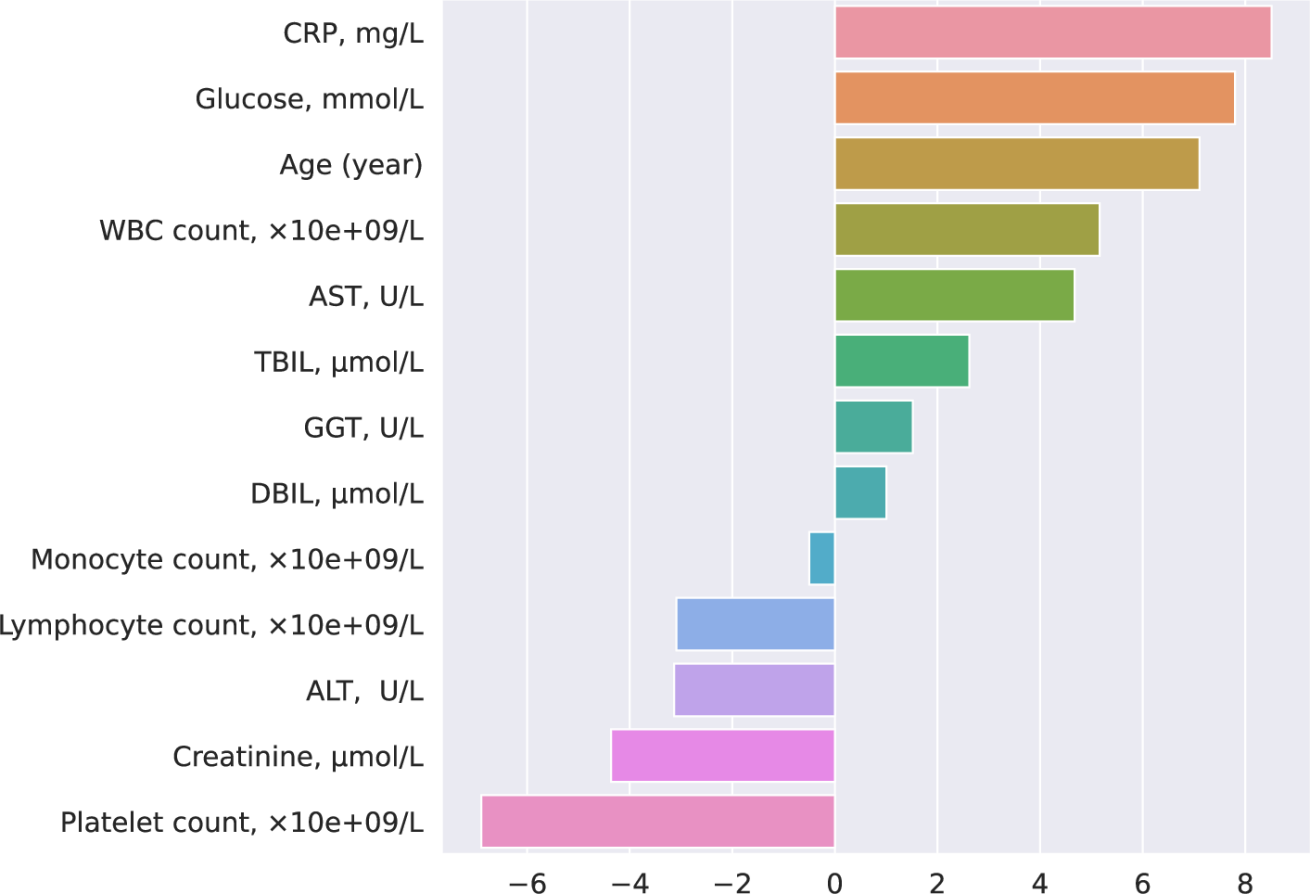
LR coefficients obtained from the biochemistry data. LR model provides direct access to the computed coefficients and summarizes information about the contribution of each feature in the final output. The positive or negative coefficient values indicate an increased or decreased weight passed to the logistic function, which results in obtaining a positive or negative answer, respectively. In our analysis of the disease progression, severe cases were labeled as positive. This allows the identification of the biochemical features related to case severity. To generate compatible coefficient values for parameters with different ranges (e.g. lymphocyte count typically ranges from 0 to 3 while CRP ranges from 0 to 200) we normalized the data using the l2 normalization.

**Fig 6:**
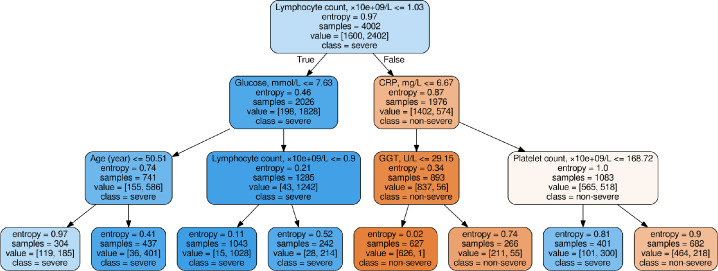
Results from the DT model trained on the biochemistry data. The DT is a directed graph based model which consists of nodes, leaves and edges. Each node contains a decision with two output edges for True and False rule, with the exception of the terminal nodes, which are called leaves and denote the class label. For each sample we can manually move from the root to a leaf by checking each node’s rules. As this model is prone to overfitting, we limited the depth of the tree to 3 and the minimum number of samples in the leaf to 120 (5% of the larger class). This approach allows monitoring the most stable and important decision rules which distinguish severe and non-severe cases.

The LR and DT ML models integrated within the COVIDomic platform were trained on the biochemistry dataset. It is important to emphasize that synthetic values cannot be used for the evaluation of the models, as the training and test sets would have been generated on the same original samples. As such, we did not evaluate the quality of the predictions, but rather focused on the identification of the most important features (Figs 5 and 6).

As shown on Fig 5, C-Reactive Protein (CRP) (a non-specific acute-phase protein induced by IL-6 in the liver), age and the glucose level are positively correlated with an increased risk of developing a more severe form of the disease, while platelet count, creatinine, and lymphocyte count are the most negatively correlated features. These results are in agreement with previous studies [20,44,45]. For instance, high expression of CRP is associated with bacterial or viral infections and was also reported to be significantly correlated with an increased probability of developing the severe form of the disease [46]. CRP level was significantly higher in the early stages of severe cases compared to mild cases [47,48]. As emphasized in previous studies [49–51], glucose level also affects the severity of the SARS-CoV-2 infections. It was shown that increased glucose level in blood is associated with a poorer prognosis in COVID-19 patients [52,53], and patients with type 2 diabetes had a significantly higher mortality than non-diabetic individuals [52]. Our analysis revealed that higher blood levels of liver function biomarkers (such as AST, GGT and TBIL), positively correlate with COVID-19 severity, which aligns with recently published evidence showing that impaired liver function is associated with poorer clinical outcomes in patients with COVID-19 [54,55]. However, another study reported that the levels these enzymes were not significantly different between COVID-19 patients and individuals hospitalised with community-acquired pneumonia [56], suggesting that future studies using larger patients cohorts are required to better define the link between markers of the liver function and severity of the COVID-19 disease.

As shown on Fig 6, our analysis revealed that absolute lymphocyte count shows the best threshold value for entropy reduction. In line with this observation, it was recently reported that lymphocyte count is often decreased in severe COVID-19 cases [57,58].

It was found that patients who succumbed to the disease had an increased white blood cell (WBC) count, suggesting that in severe cases, a significant increase of the WBC count is associated with a worse diagnosis and poor outcome [59]. Furthermore, subsequent studies statistically supported that an elevated WBC count was strongly associated with the aggravation of the disease in COVID-19 patients [60]. The increase of the WBC count is driven by elevated neutrophils, as decreasing trends were observed for lymphocytes, monocytes and eosinophils. WBC count, lymphocytes, monocytes and eosinophils were recently identified as biomarker abnormalities in COVID-19 patients with severe systemic disease [61].

### Feature importance analysis based on viral genome data

SARS-CoV-2 genomes were obtained from the Global Initiative on Sharing All Influenza Data (GISAID) (https://www.gisaid.org/). The initial GISAID dataset was not labeled in terms of disease severity, and contained manual assessments from clinicians without proper standards and formatting allowing automatic classification. In our analysis, patients which required hospitalization or died from COVID-19 were defined as severe cases, whereas asymptomatic individuals or symptomatic patients that did not require admission were labeled as non-severe cases. Patients with “unknown” hospitalization status or without information about the disease progression and severity were removed from the dataset, resulting in a dataset of 2402 severe and 1600 non-severe cases with available SARS-CoV-2 genome data. All viral sequences were aligned with the Genome Reference Sequence (NC_045512) reference genome and reference multiple sequence alignment was performed to find the coordinates of each variable site in the sequences. A total of 7463 variable sites were identified. In this analysis, the 5’ untranslated region (UTR) and 3’UTR coordinates were excluded [61], as these regions are highly noisy and unlikely to contain relevant information for predicting the infection severity. However, the UTR region harbors RNA structural elements that play a critical role in transcription initiation and replication [62,63], and therefore may be included into the analytical pipeline as our platform evolves. Following filtering, we obtained a set of 7150 unique variable sites across all cases. It is worth emphasizing that the prediction of the severity of COVID-19 disease using the viral genome is more reliable when done at the early stages of the disease progression, as the viral genome rarely changes in a patient (the median number of intra-host variants is 4 [33]). Therefore, the analysis can be carried out using samples collected before symptoms development.

**Fig 7:**
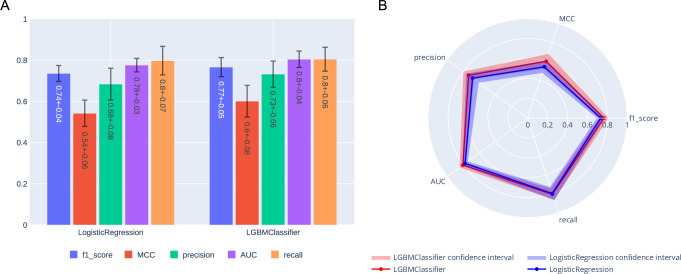
Scores obtained with the two best models: LGBM and LR. Bar plot (A) and radar plot (B) are shown. Results were obtained using 5-fold 20% cross validation. LGBM obtained a f1 score of 0.77, compared to 0.74 for LR.

**Fig 8:**
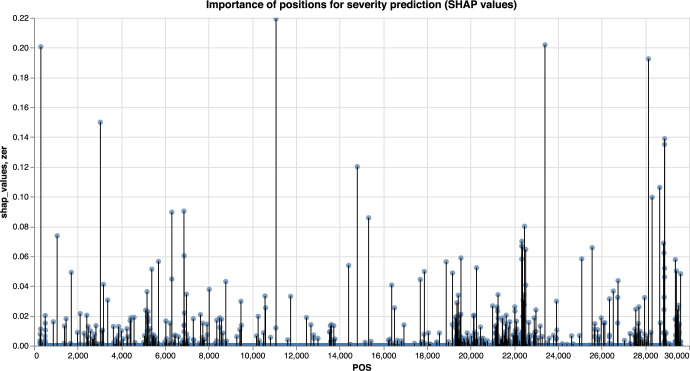
Feature importance analysis using SHAP method. Manhattan plot. Feature importance based on the LGBM model, computed using the SHAP method.

**Fig 9:**
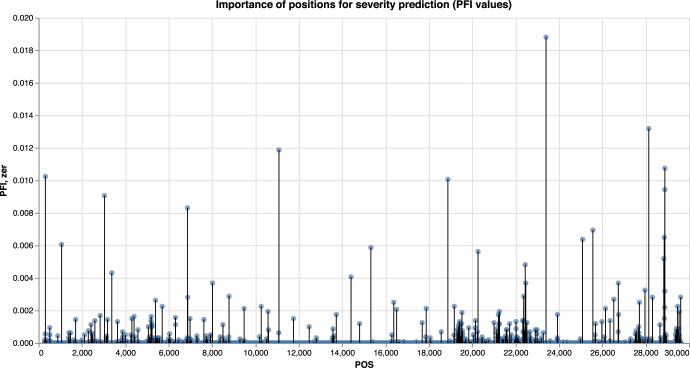
Feature importance analysis using PFI method. Manhattan plot. Feature importance based on the LGBM model, computed using the PFI method.

**Fig 10:**
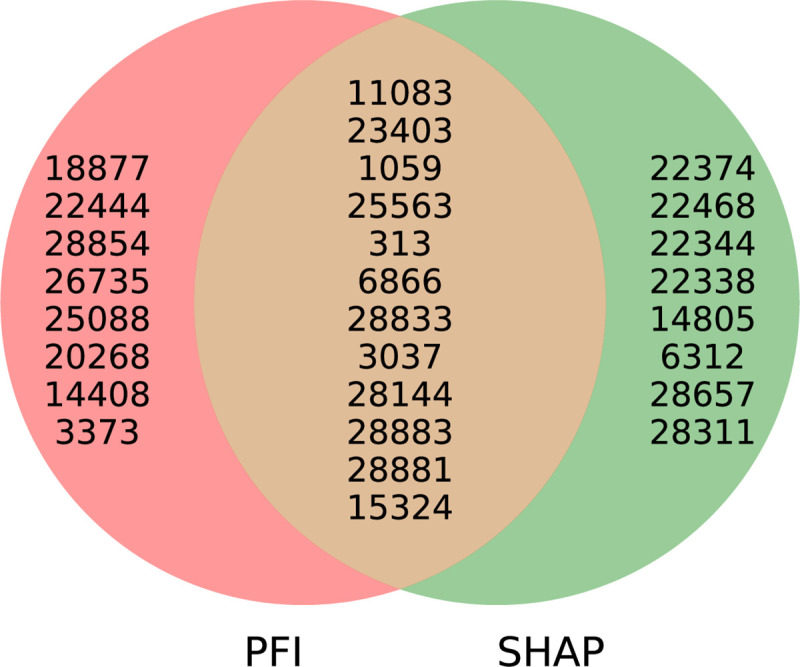
Analysis of the predicted features common to the PFI and SHAP methods. The top 12 mutations predicted by both methods are depicted (See Table 1 and main text for details).

The LGBM and LR models were trained to distinguish between severe and non severe COVID-19 cases, with the LGBM model slightly outperforming the LR for most of the metrics (Fig 7). The results of the feature importance analysis using the SHAP and PFI methods are displayed on Figs 8 and 9 respectively. Features with the highest importance scores obtained using the PFI and SHAP methods are summarized on Fig 10. Specifically, all positions were ranked according to their importance for severity prediction by two methods, the top 20 positions with highest PFI or SHAP values were selected and 12 intersecting positions predicted by both algorithms are summarized in the Venn diagram (Fig 10) and Table 1. Values of the LR coefficients and LGBM feature importance were also computed. For each fold, feature importance was calculated and then averaged over all folds. For computing feature importance based on the SHAP method, we average the absolute Shapley values per feature over the data, so that features with large value are more important for the severity prediction model. The SHAP Tree Explainer and the Linear Explainer were applied to compute the LGBM and LR feature importance, respectively. For this experiment, the default LR scoring metric, accuracy, was used to evaluate the model performance during the feature permutation.

**Table 1:** Top 12 overlapping Genomic features and amino-acid changes obtained with the PFI and SHAP methods. (* Amino acid position in ORF1ab polyprotein is provided).

Genomic position	Gene name	Protein name	Amino Acid
313	ORF1ab	Nonstructural protein NSP1	16L*
1059	ORF1ab	Nonstructural protein NSP2	265T*
3037	ORF1ab	Nonstructural protein NSP3	924F*
6866	ORF1ab	Nonstructural protein NSP3	2201N*
11083	ORF1ab	Nonstructural protein NSP6	3606L*
15324	ORF1ab	Nonstructural protein NSP12	5020N*
23403	S	Spike glycoprotein	614D
25563	ORF3a	Nonstructural protein NS3a	57Q
28144	ORF8	Nonstructural protein NS8	84L
28833	N	Nucleocapsid protein	187S
28881	N	Nucleocapsid protein	203R
28883	N	Nucleocapsid protein	204G

Although genomes of SARS-CoV-2a and SARS-CoV-2g strains identified in Europe present a higher mutation frequency compared to the unique Asian variant they have originated from [64], SARS-CoV-2a has spread in countries with relatively low COVID-19 cases, whereas SARS-CoV-2g is more common in highly affected countries. Interestingly, the difference in pathogenicity between these two strains is based on mutations in the 3 nucleotides block located within the positions 28881–28883 of the SARS-CoV2 genome region. Notably, mutations at this region were among the key overlapping mutational features detected by the COVIDomic platform importance analysis (Table 1). While SARS-CoV-2g has the sequence GGG in the positions 28881–28883, this sequence is mutated to AAC within the SARS-CoV-2a genome. The 28881–28883 region encodes the SARS-CoV-2 nucleocapsid (N) protein, which intervenes with the replication and affects host cell responses against viral infection, including cell cycle regulation and immune response [65]. Studies suggest that the (GGG>AAC) mutation negatively affects the N protein. This would explain why COVID-19 cases are less severe in areas where SARS-CoV-2a strain is predominant [66]. If the (GGG>AAC) block mutation contributes to reducing SARS-CoV-2a pathogenicity, then 203–204:RG>KR protein position could be used as a target for designing therapies aiming at reducing viral replication, and subsequently the pathogenicity of SARS-CoV-2 infection [66].

On the other hand, mutations at the position 23403 (D614G change in viral protein S) could be associated with the increased transmission rate and higher COVID-19 patients mortality [67–70]. There are several hypotheses underlying this observation. It was reported that structural changes induced by the D614G mutation might either facilitate the shedding of S1 from viral-membrane-bound S2, or impact RBD-ACE2 binding [71]. Alternatively, the D614G mutation may impact the virus transmission and severity of the disease via long term B-cell memory response inhibition [71,72], although further confirmation of these hypotheses is warranted in larger patients cohorts.

Another example of a clinically informative aberration detected by our platform is the mutation located at the position 25563 in the ORF3a gene. In SARS-CoV-2, this gene is implicated in viral release, inflammasome activation, and cell death, while its deletion reduces viral titer and morbidity in animal models [73], suggesting that it could provide a promising target for novel therapeutics against COVID-19 disease.

Furthermore, mutation at position 28144 (resulting in a Ser/Leu change), is predicted to impact the structural integrity of the protein [74], although its effect of the severity of the disease remains to be elucidated.

Taken together, this analysis demonstrates that it is possible to extract biologically important features (positions), which influence the disease severity, using ML models. While ML techniques were able to automatically identify clinically relevant mutations that were previously discussed in the literature, we were able to highlight additional potentially informative mutations which have not yet been reported. As such, the use of ML-based approaches allows a faster response to the emergence of potentially dangerous mutations within the viral genome.

### Risk factors analysis based on metatranscriptome data

While COVIDomics platform has the capability to perform risk factors analysis based on metatranscriptome data, at the time of manuscript preparation, we were able to find only one recently published dataset containing metatranscriptome with 8 severe and 23 non-severe cases samples left after quality filtration step [33]. Due to the limited number of currently available samples, we could not generate additional synthetic data with a realistic feature distribution required for the risk factor analysis. As such, we could not include the metatranscriptome risk factor analysis to the case study pipeline. However, this functionality is integrated within the platform, and as more datasets will become available, assessing the spectrum of microorganisms in the metatranscriptome and analyzing the resistance of microorganisms to antimicrobial agents may allow a more accurate prediction of the COVID-19 severity, and assist with adjusting a therapeutic approach.

### Feature importance analysis and disease severity prediction on combined data types

**Fig 11:**
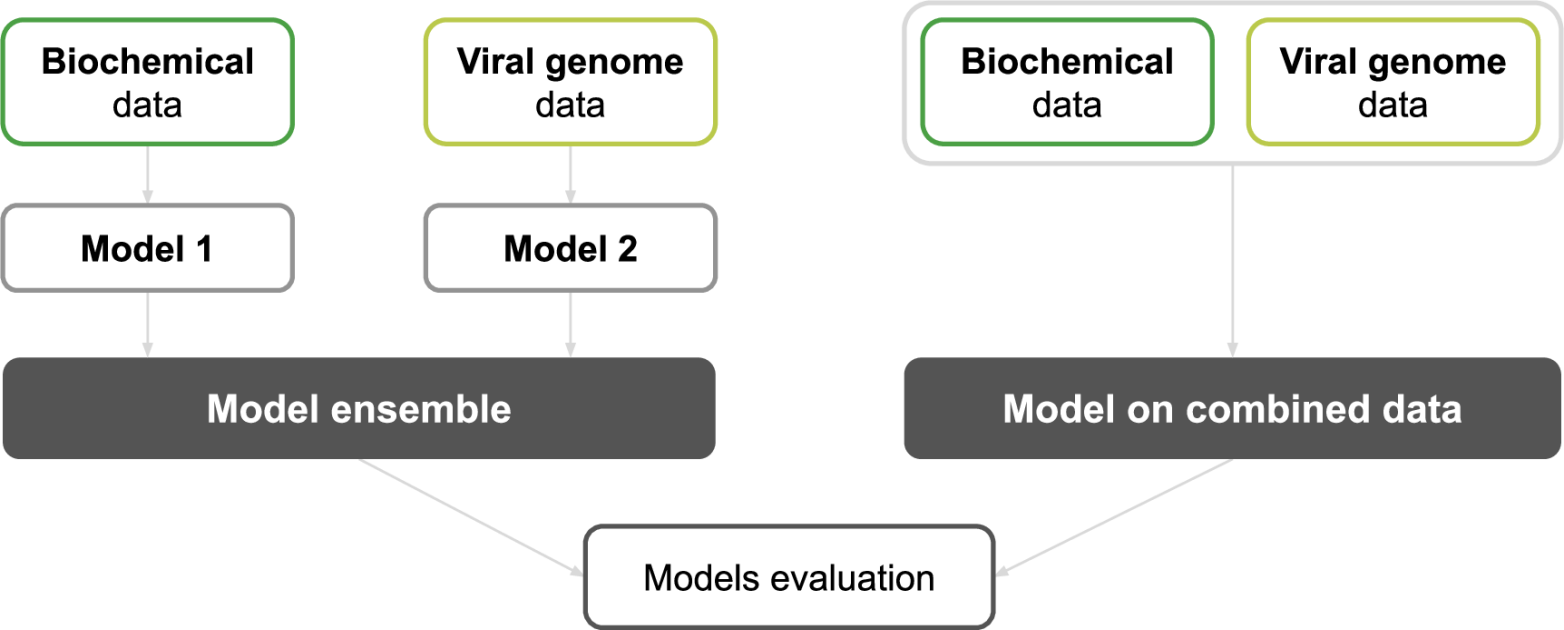
Description of approaches for building models on different data types. Two different approaches were considered for combining two types of data. (Left) The first method builds a model ensemble by combining two models trained separately on the two types of datasets (biochemistry data and viral genome data). (Right) The second approach is based on training a new model on a dataset made of both data types combined together.

Conducting an analysis of COVID-19 severity using multiple data types has pros and cons. On one hand, training a single ML model on the combined dataset, allows computing the PFI and SHAP values for all features (Fig 11), providing an overview of the relative importance of features extracted from different data types. However, on the other hand, this approach does not take into account that the different data types are not necessarily available at the same time. An alternative approach would be to proceed with prediction once the first type of data is available. The accuracy of this first prediction analysis can be incrementally improved as additional data sources become available. Such methodology can be implemented through the use of a model ensemble (Fig 11).

**Fig 12:**
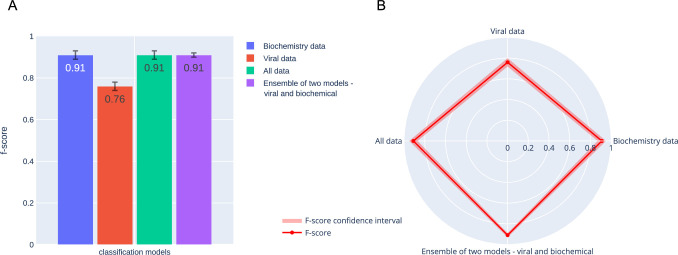
Results for the training of models on various types of data. Bar plot (A) and Radar plot (B) are sown. f1 scores obtained for four models designed to distinguish severe from non-severe COVID-19 cases. The first model was trained solely on biochemistry data, the second one on viral genomic data only, the third one was trained on a combined data sets made of biochemistry and viral data, while the last one is a model ensemble obtained by combining two models trained separately on the two types of datasets (biochemistry data and viral genome data). The data were merged and the model ensemble was designed using the soft voting function implemented in sklearn. Synthetic (oversampled) biochemistry data were used to increase the size of the biochemistry dataset used alongside the experimental viral genomic data.

**Fig 13:**
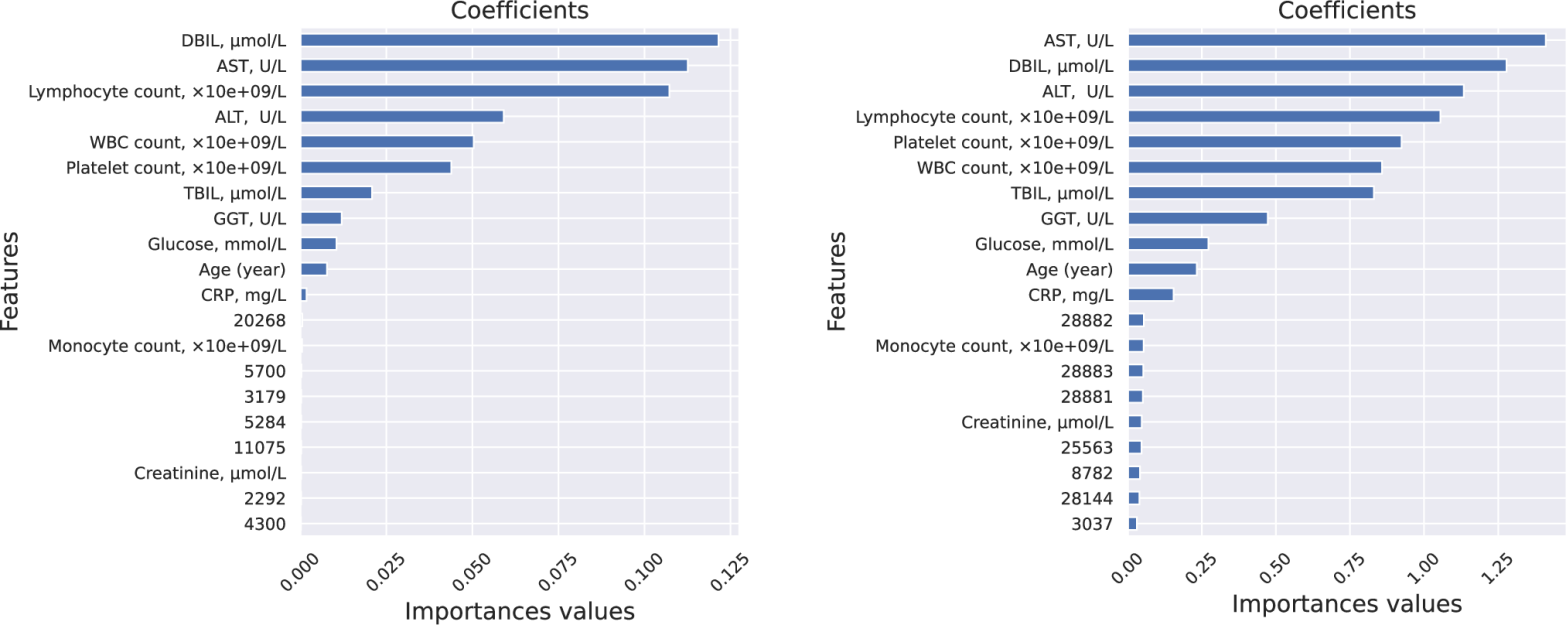
PFI and SHAP values for the feature importance analysis. The features from the biochemistry data have significantly higher scores than the features from the viral genome data for both approaches. The biochemistry data were generated separately for severe and non-severe cases, and therefore could have been identified by the model as most important.

In this framework, there is a separate model for each data source. The size of the model ensemble is not restricted, and the number of models corresponds to the number of different data sources to be taken into account for the predictions. The models can be designed and the features of different data types weighted so that the final ensemble model is capable of exploiting all features effectively. The weights can also be designed to favor models which demonstrate better predictive power and accuracy. This flexible approach allows integration of the new data types which are corresponding to a new clinical analysis. However, the model ensemble does not take into account the possible relationships between different types of clinical parameters. If the data is originating from different sources, the ensemble will not be able to find dependencies that may be important for predicting the severity of COVID-19 disease. For example, a particular SARS-CoV-2 mutation (a novel m^6^ A methylation loci in the S protein of SARS-CoV-2) was recently shown to increase the risks of developing gastrointestinal (GI) symptoms in COVID-19 patients [50]. This interplay could not be identified with a model ensemble made of a symptom-based model and a viral genome-based model, as each model in the ensemble is trained independently on one type of data (mutation or GI symptoms) to identify how features extracted from a given type of data influences the severity of the disease. These models provide conclusions based on two types of features, mutations and GI symptoms, independently, while the model trained on combined data can take into account correlated features between these different data types. For such cases, building a model that combines all data into a single dataset allows the ML classifiers to identify and exploit the dependencies between the features extracted from different types of data (Fig 12 and Fig 13).

Given the specific situation, one approach may be preferred over the other, especially when multimodal data (clinical, laboratory and genomics) is used. One example is when there are pre-calculated values for fixed types of data, such as if a hospital has a standard set of COVID-19 patient measures. In this case, the datasets are uniform, and thus, using one large model is more effective. Indeed, combined standardized patient data can be treated most effectively using a single model. Another scenario to consider is when the data sources are unknown or may change over time. For instance, a patient may come to the hospital with results obtained from the specialized laboratories or the hospital itself may change the number and types of diagnostic and prognostic procedures depending on the symptoms. In that case, using a model ensemble would be more appropriate. Using a model ensemble is also preferable when data of any source is dynamically changing, as only one model has to be updated regularly to get more accurate results.

## Availability and future directions

Since the outbreak of the COVID-19 pandemic, several initiatives have been launched to provide the scientific community with online tools and databases to accelerate the COVID-19 research. For instance, the COVID-19 data portal (https://www.covid19dataportal.org/) provides access to viral and host sequences, as well as proteomic and biochemistry data. Researchers from the Centre for Genomic Regulation (CRG) launched in April 2020, as part of the wider European COVID-19 Data Platform, a database (https://covid.crg.eu) which brings together standardized datasets for sharing and analysis. Web services have been developed to offer accessible computational tools for the standardized analysis of the large amount of diverse health data produced during the last several months [75]. While promising, many of these tools are designed for the analysis of specific data types. For example, the Global Evaluation of SARS-CoV-2 Sequences (https://wan-bioinfo.shinyapps.io/GESS/) is a resource that provides a comprehensive analysis based on high-quality complete SARS-CoV-2 genomes. However, tools allowing an integrative analysis of a combination of data of various types have higher potential to bring valuable insights into the complex mechanisms underlying the disease evolution [76].

The COVIDomic platform (available at http://covidomic.com/) offers the first integrated and user friendly analytical pipeline allowing biological scientists from both academia and pharmaceutical industry to make the best use of all the available data typed associated with COVID-19. Although this article describes the most current version of the platform, a COVIDomic toolset is continuously updated, and newly released data collections related to either COVID-19 or other non-COVID-19 bacterial and viral infections will be uploaded routinely. Furthermore, predictive capabilities of the models incorporated into our platform, although optimal, will be dynamically re-evaluated through a continuous learning system, which will allow integrating the best ensemble of predictive models as more data become available.

With an increased awareness of potential future pandemics, this type of integrated multi-modal analytical pipeline for complex viral infection could be used in the context of other epidemics caused by pathogens.

## Supporting information

S1 Fig Schematic representation of the workflow of the platform with its four components. Component 1 is the processing pipelines. It focuses on abstraction over memory, disk and CPU-intensive data processing tasks that support scalability. The component 1 also processes raw FASTQ metatranscriptomic data into human gene-level read count data, bacterial and viral taxon-level read count data, and antibiotic resistance gene-level read count data. The component 2 is the data storage and management system. It manages the pipeline tasks, input and output file processing and data download from public resources. Component 3 is the web server with the REST API. It interacts with the data storage and management system, manages user registration and access restrictions and provides the results of the analysis via secure API. Component 4 is a web application with the user interface. It allows the users to register, login and upload data securely, view user data processing status and perform the analysis on combined public and private data.
(TIF)

S1 Text Technical description of the platform capabilities and their implementation. **Table A.** Description of the main programs included in the full processing pipeline of the COVIDomic platform.
(DOCX)
