# Supplementary figures and images for: COVIDomic: A multi-modal cloud-based platform for identification of risk factors associated with COVID-19 severity
Source: PLoS Comput Biol. 2021 Jul 14;17(7):e1009183. doi: 10.1371/journal.pcbi.1009183 (PMC8312936; doi:10.1371/journal.pcbi.1009183)

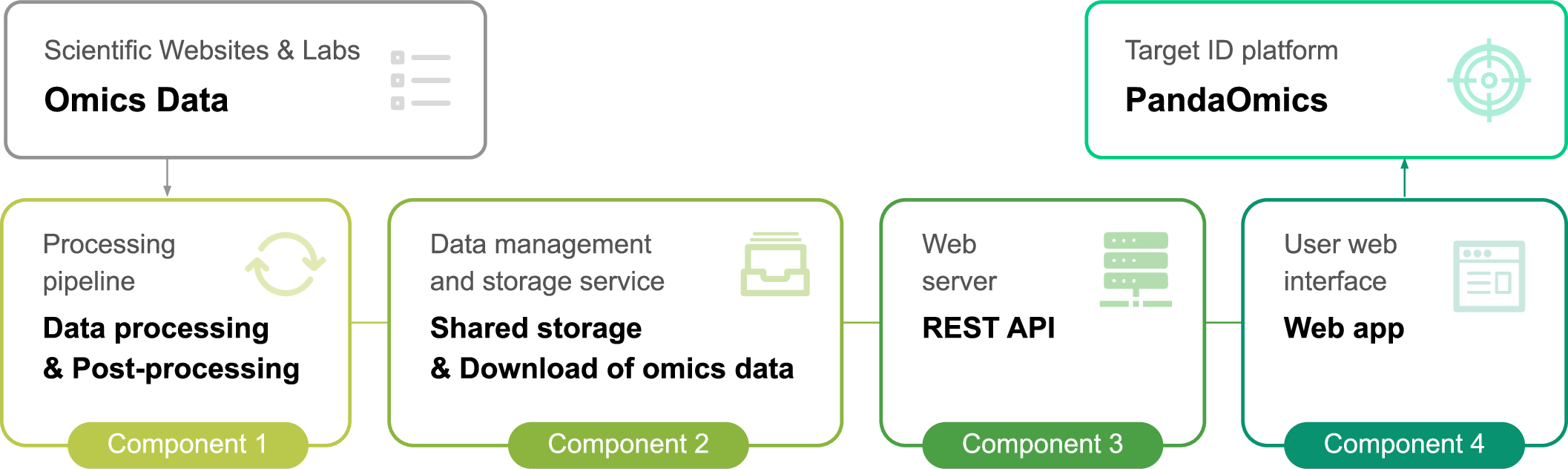

Supplement: S1 Fig — Component 1 is the processing pipelines. It focuses on abstraction over memory, disk and CPU-intensive data processing tasks that support scalability. The component 1 also processes raw FASTQ metatranscriptomic data into human gene-level read count data, bacterial and viral taxon-level read count data, and antibiotic resistance gene-level read count data. The component 2 is the data storage and management system. It manages the pipeline tasks, input and output file processing and data download from public resources. Component 3 is the web server with the REST API. It interacts with the data storage and management system, manages user registration and access restrictions and provides the results of the analysis via secure API. Component 4 is a web application with the user interface. It allows the users to register, login and upload data securely, view user data processing status and perform the analysis on combined public and private data. (TIF) [file pcbi.1009183.s001.tif]
