# Supplementary material for: COVIDomic: A multi-modal cloud-based platform for identification of risk factors associated with COVID-19 severity
Source: PLoS Comput Biol. 2021 Jul 14;17(7):e1009183. doi: 10.1371/journal.pcbi.1009183 (PMC8312936; doi:10.1371/journal.pcbi.1009183)
Supplement: S1 Text — Table A. Description of the main programs included in the full processing pipeline of the COVIDomic platform. (DOCX) [file pcbi.1009183.s002.docx]

**COVIDomic: a multi-modal cloud-based platform for identification of risk factors associated with COVID-19 severity**

**S1 Text.**

**Technical description of the platform capabilities and their implementation**

1 COVIDomic data collection

In the current version, COVIDomic uses open community data from more than 100 experiments obtained from the Sequence Read Archive (SRA) ([https://www.ncbi.nlm.nih.gov/sra](about:blank)) and the NGDC 2019 Novel Coronavirus Resource (2019nCoVR) ([https://bigd.big.ac.cn/ncov/?lang=en](about:blank)), which contains over 30 000 sequences of the SARS-CoV-2 genome. A collection of annotated and analysed pulmonary transcriptomes from patients with different types of diseases (including fibrosis, pneumonia, etc.) was processed by the COVIDomic pipeline to be used as a point of reference for singular metatranscriptome analyses.

2 Technical implementation

The COVIDomic platform comprises four main components (Fig S1), namely, the metatranscriptomic data processing pipeline, the data storage and management system, web server with the Representational State Transfer (REST) Application Programming Interface (API), and the web application which includes the user interface and display of the analysis results. The processing pipeline includes a set of omics data processors integrated into a common system. The pipeline configuration uses two servers and a common file storage. The implementation supports horizontal scaling, which allows increasing the throughput of the entire pipeline as a function of the amount of data to be managed. The scaling is implemented by adding preprocessing and postprocessing servers and can be performed in real time (i.e. does not require any interruption of the workflow). The data management and storage service monitors the data processing pipelines, the task flow, it tracks the results of the analysis and provides access to the output files generated by the pipeline. The web server with REST API supports user management, secure access to the files, provides reports summarizing task status, and gives access to the data used in the web application. The web application allows researchers to register as users and upload their metatranscriptomics data to the processing service. The users can also use the results of the taxonomic analysis in conjunction with the publicly available data. The web application provides a convenient approach for the qualitative comparison of sample groups using public and private datasets. The approach is based on dimension reduction analysis (DRA) and selects arbitrary groups of samples on a DRA plot using a simple lasso select interface to compare the abundance of taxons in different sample groups. A separate section is dedicated to quality control summary for all user-provided and public data samples.

The COVIDomic platform runs on Intel’s 2nd Generation Intel Xeon Scalable Processors, formerly Cascade Lake, with built-in Intel Deep Learning Boost and delivers high-performance inferencing for AI workloads, along with diverse sets of workloads handling the massive datasets and near-real-time analytics intrinsic to COVIDomic. Highly optimized, threaded, and vectorized math functions, such as those required for PCA analysis, were performed using Intel Math Kernel Library (MKL).

*2.1 Full processing pipeline*

The full processing pipeline includes a set of nine program blocks that are executed sequentially, with the possibility of parallel launch (see S1 Table). The workflow of the pipeline is executed through several servers to achieve an optimal and stable operation of all programs. The preprocessing programs have high requirements for server hardware configuration, while post-processing programs are less resource intensive. Web server configuration may have low hardware configuration requirements.

| **Program** | **Reference** | **Short description** | **Input** | **Output** |
| --- | --- | --- | --- | --- |
| Custom script | Insilico Medicine | Downloading data from the user | - | (Meta-) transcriptome fastq file(s). |
| FastQC | [https://www.bioinformatics.babraham.ac.uk/projects/fastqc/](about:blank) | Creating a report on the number and the quality of reads for each sample | (Meta-) transcriptome fastq file(s). | Sequencing quality report (HTML) |
| BWA | [http://bio-bwa.sourceforge.net/](about:blank) | Alignment of reads with the human transcriptome, filtering non-human reads | (Meta-) transcriptome fastq file(s). | Non-human reads fastq |
| FastQC | [https://www.bioinformatics.babraham.ac.uk/projects/fastqc/](about:blank) | Creating a report on the number and the quality of reads for non-human data | Non-human reads fastq | Non-human reads quality report (HTML) |
| Kraken 2 | [[1]](about:blank) | Analyzing the presence of different microorganisms in the metatranscriptomic data | Non-human reads fastq | Kraken report |
| BWA, SAMtools | [http://bio-bwa.sourceforge.net/](about:blank)  [http://www.htslib.org/doc/samtools.html](about:blank) | Alignment of reads with the CARD database. | Non-human reads fastq | Antibiotic resistance BAM |
| BWA, SPAdes, quast | [http://bio-bwa.sourceforge.net/](about:blank)  [http://cab.spbu.ru/software/spades/](about:blank)  [http://cab.spbu.ru/software/quast/](about:blank) | *De novo* assembly of the SARS-CoV-2 genome | Non-human reads fastq | SARS-CoV-2 genome (fasta) |
| MAFFT, SINA, custom scripts | [https://mafft.cbrc.jp/alignment/software/](about:blank)  [https://github.com/epruesse/SINA](about:blank) | SARS-CoV-2 phylogeny reconstruction | SARS-CoV-2 genome (fasta) | SARS-CoV-2 phylogenetic tree |
| Custom scripts | Insilico Medicine | Visualizing the results | Kraken report | Bacterial abundance analysis |
|  |  |  | Antibiotic resistance BAM | Antibiotic resistance analysis |
|  |  |  | Patients biochemical parameters and age, aligned SARS-CoV-2 genomes | Risk factors analysis |

**Table A.** Description of the main programs included in the full processing pipeline of the COVIDomic platform.

*2.2 Preprocessing server hardware configuration*

The pre-processing server includes three components (see S1 Table): a program for interacting with the REST API and for the processing and downloading of omics data from external resources (HTTP/FTP), a program for reporting sample quality control, and a program for the alignment of reads with the human genome reference.

*2.3 Hardware configuration of the post-processing server and report*

The post-processing server includes six components (see S1 Table): a program for reporting the number and the quality of non-human reads, a program for representing the abundance of various microorganisms in the metatranscriptome, a program for analyzing the resistance of microorganisms to antimicrobial agents, a program for the *de novo* assembly of the SARS-CoV-2 genome, a program for risk factor analysis based on various data, and a program for displaying the results and synchronizing files with a REST API server.

*2.4 Web server hardware configuration*

The web server provides two types of services, the REST API and the management and interaction service. The web server interacts with the bioinformatics web service PandaOmics ([https://pandaomics.com](about:blank)), a computational suite designed internally to perform the analysis of multi-omics data within a single comprehensive network biology pipeline. PandaOmics provides direct access to information ranging from disease signatures to prospective targets and drug candidates. It also combines classic bioinformatics tools such as signaling pathway analysis with an AI-powered toolkit for drug and target discovery. This multimodal approach using big data and biology allows a detailed characterization of the interplay between molecular structures, properties, alteration in biological samples and drug response.

*2.5 Architecture and job description*

The web server regularly synchronizes the report directory on the post-processing server with the REST API static files directory. When a list of samples is requested, the REST API functions are used to search the sample database and map the requested hashes to the set of available static files. Depending on the types of reports, separate groups are organized (e.g. ‘start_fastqc’, ‘fastqc’, ‘kraken’, ‘bwa’, etc.), for which the URLs are generated. This list is provided in a serialized form (JSON) to the source of the request, which can be either the management and interaction service web application, or an external online service (such as the PandaOmics platform). The database of samples can be compiled automatically by uploading the CSV files in the specified format, manually by using a form in the web interface, or by sending a POST request from an external service.

**Reference**

[1] [Wood DE, Lu J, Langmead B. Improved metagenomic analysis with Kraken 2. Genome Biol. 2019;20: 257.](about:blank)
